# A Multitechnique Study of C_2_H_4_ Adsorption on a Model Single-Atom Rh_1_ Catalyst

**DOI:** 10.1021/acs.jpcc.4c03588

**Published:** 2024-09-05

**Authors:** Chunlei Wang, Panukorn Sombut, Lena Puntscher, Manuel Ulreich, Jiri Pavelec, David Rath, Jan Balajka, Matthias Meier, Michael Schmid, Ulrike Diebold, Cesare Franchini, Gareth S. Parkinson

**Affiliations:** † Institute of Applied Physics, TU Wien, Vienna 1040, Austria; ‡ Faculty of Physics, Center for Computational Materials Science, University of Vienna, Vienna 1090, Austria; § Dipartimento di Fisica e Astronomia, Università di Bologna, 40126 Bologna ,Italy

## Abstract

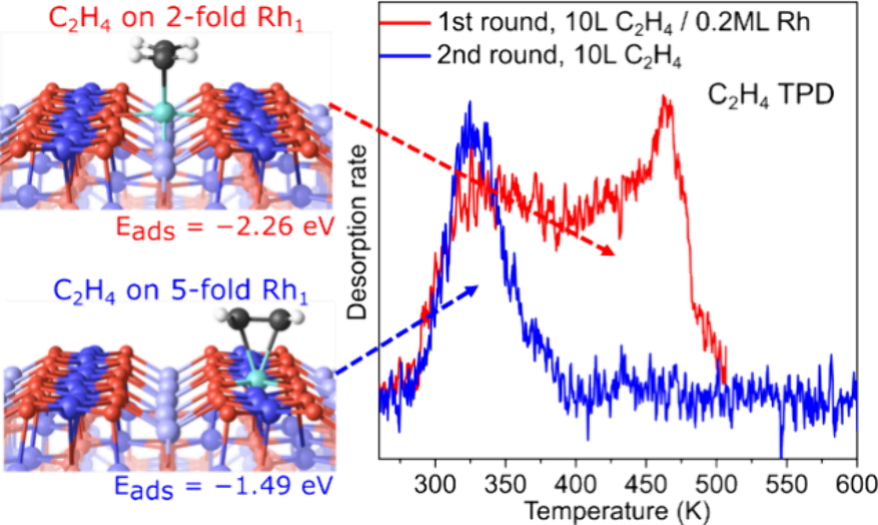

Single-atom catalysts are potentially ideal model systems to investigate structure–function relationships in catalysis if the active sites can be uniquely determined. In this work, we study the interaction of C_2_H_4_ with a model Rh/Fe_3_O_4_(001) catalyst that features 2-, 5-, and 6-fold coordinated Rh adatoms, as well as Rh clusters. Using multiple surface-sensitive techniques in combination with calculations of density functional theory (DFT), we follow the thermal evolution of the system and disentangle the behavior of the different species. C_2_H_4_ adsorption is strongest at the 2-fold coordinated Rh_1_ with a DFT-determined adsorption energy of −2.26 eV. However, desorption occurs at lower temperatures than expected because the Rh migrates into substitutional sites within the support, where the molecule is more weakly bound. The adsorption energy at the 5-fold coordinated Rh sites is predicated to be −1.49 eV, but the superposition of this signal with that from small Rh clusters and additional heterogeneity leads to a broad C_2_H_4_ desorption shoulder in TPD above room temperature.

## 1 Introduction

Supported single-atom catalysts (SACs) have garnered much attention due to their cost efficiency, catalytic activity, and high selectivity for various reactions. Significant efforts have been devoted to understanding the reaction mechanisms in single-atom catalysis,^1−5^ but providing a clear elucidation of the structure–activity relationship is complicated by the inability to determine the local bonding environment of the active atoms. Moreover, the stability of the active centers during reactions is still a subject of debate, and it is difficult to determine whether small clusters form under reaction conditions.^6−10^ Traditionally, researchers have utilized model catalysts based on well-defined single crystals under ultrahigh vacuum (UHV) conditions to study structure–property relationships in catalysis^11−14^ However, few model systems exist in which single atoms remain stable on model supports at reaction temperatures. To date, most model studies of SAC have been limited to the adsorption and reaction of inorganic molecules such as CO, O_2_, and water.^15−17^ Here, we select a representative olefin, ethylene (C_2_H_4_), and study its interaction with a model Rh/Fe_3_O_4_ catalyst featuring single atoms in various configurations.

Olefins serve as vital reactants in many industrial processes, such as polymerization or hydroformylation of aldehyde synthesis. A clear understanding of these catalytic reaction pathways is important for the promotion of the catalytic properties. Recently, Guo et al. reported an in situ visualization of ethylene polymerization by scanning tunneling microscopy (STM) on a carburized iron model catalyst, which provides a direct evidence for this growth process.^18^ The hydroformylation reaction is typically performed homogeneously in solution utilizing Wilkinson’s catalyst, but recently, oxide-supported Rh single-atom catalysts have been shown to exhibit remarkable catalytic performance.^19−21^ An important aspect of this reaction is the coadsorption of CO and C_2_H_4_, so an atomic-scale understanding of the C_2_H_4_ adsorption behavior on Rh_1_ would be timely.

In this paper, we utilize a single-crystal Fe_3_O_4_(001) support that can stabilize a variety of different metals and where the coordination can be tuned by the preparation conditions.^22,23^ Surface-sensitive techniques such as STM, temperature-programmed desorption (TPD), and X-ray photoelectron spectroscopy (XPS) are employed to explore how ethylene interacts with Rh in different configurations. We found that ethylene adsorption on 2-fold Rh_1_ sites leads to the formation of a pseudosquare planar structure, in which the Rh relaxes toward the support forming a weak coordination with subsurface oxygen. When the sample is heated in a TPD experiment, ethylene desorption is accompanied by the evolution of Rh adatoms to a substitutional cation geometry in the support. Desorption from the 5-fold coordinated Rh sites occurs just above room temperature, comparable to desorption from small Rh clusters.

## 2 Experimental and Computational Methods

### Experimental Section

The experiments were performed on natural Fe_3_O_4_(001) (6 × 6 × 1 mm) single crystals purchased from SurfaceNet GmbH. All the samples were cleaned by cycles of sputtering (10 min, 1 keV Ar^+^ STM chamber or Ne^+^ TPD/XPS chamber) and annealing (923 K, 20 min). In the final cleaning cycle before measurement, the sample was oxidized by annealing in 2 × 10^–6^ mbar O_2_ for 20 min at 923 K. Oxidative annealing in oxygen leads to the growth of new pristine surface layers and yields the reconstructed (√2×√2)R45° surface.^24^ Rh atoms were deposited using an e-beam evaporator (FOCUS), with the flux calibrated using a temperature-stabilized quartz microbalance (QCM). One monolayer (ML) is defined as one Rh atom per Fe_3_O_4_(001)-(√2×√2)R45° surface unit cell, which is equivalent to 1.42 × 10^14^/cm^2^.

Two separate UHV systems were utilized during this work: Imaging experiments were performed in a setup that includes a coupled preparation chamber (base pressure *p* < 10^–10^ mbar) and analysis chamber (*p* = 5 × 10^–11^ mbar). STM was performed in the analysis chamber using a μ-STM at room temperature in constant-current mode using an electrochemically etched W tip. The sample was always positively biased, meaning that empty states were imaged. The analysis chamber is also equipped with a nonmonochromatic Al Kα X-ray source and a SPECS Phoibos 100 analyzer for XPS analysis. XPS data acquired here were utilized as a fingerprint to ensure that the sample prepared in the TPD experiments was the same.

The TPD and XPS experiments were conducted in another UHV system optimized to study the surface chemistry of model catalysis.^25^ The Fe_3_O_4_(001) sample was mounted on a Ta backplate with a thin gold sheet in between to improve the thermal contact. The sample is cooled by a liquid-He flow cryostat (base temperature of ∼40 K) and is heated by the resistive heating of the Ta backplate. The vacuum system is equipped with a home-built molecular beam source, which delivers reactants with a calibrated flux (equivalent to the impingement rate at 2.66 × 10^–8^ mbar) and a top-hat profile to the sample with a 3.5 mm diameter.^25,26^ We give gas doses in Langmuir units, and 1 L is defined as 1 × 10^6^ torr s. The Rh was deposited using the same procedure as in the STM experiments described above. C_2_H_4_ was used for the TPD experiments. A quadrupole mass spectrometer (Hiden HAL 3F PIC) is used in a line-of-sight geometry for TPD experiments, analyzing the desorption signal at mass 27, not 28 for C_2_H_4_, to avoid any background from trace levels of CO and N_2_ in the chamber. A monochromatized Al/Ag twin anode X-ray source (Specs XR50 M, FOCUS 500) and a hemispherical analyzer (Specs Phoibos 150) are used for XPS measurements. A grazing angle of ≈71° was used for collection of XPS spectra. A complete description of the TPD/XPS chamber design is provided in ref (25).

### Computational Details

The Vienna *ab initio* Simulation Package (VASP) was used for all DFT calculations,^27^ using the projector augmented wave method to handle the near-core regions.^28,29^ The plane-wave basis set cutoff energy was set to 550 eV. The calculations were performed using the generalized gradient approximation method with the Perdew–Burke–Ernzerhof (PBE) functional to describe electronic exchange and correlation.^30^ Dispersion terms are included according to the D3 Becke–Johnson method.^31^ An effective on-site Coulomb repulsion term U_eff_ = 3.61 eV was used for the 3d electrons of the Fe atoms.^32,33^ Although the binding energies calculated using the PBE+U functional may be less accurate compared to those obtained with more advanced methods (Table S1), we consider the accuracy of PBE+U to be acceptable. The computational cost is significantly lower with PBE+U, making it a more efficient choice for our study. The convergence criterion was an electronic energy change of 10^–6^ eV per step and forces acting on ions smaller than 0.02 eV/Å. Calculations were performed with the experimental magnetite lattice parameter (*a* = 8.396 Å) using an asymmetric slab with 13 planes (7 planes with octahedral Fe and 6 with tetrahedral Fe; the bottom 9 planes are fixed and only the 4 topmost planes relaxed) and using the Γ-point only for a large (2√2 × 2√2)R45°supercell. The slabs were separated by a 14 Å vacuum layer. The average adsorption energy of adsorbed C_2_H_4_ molecules on a Rh adatom is computed according to the formula
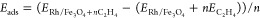
where *E*_Rh/Fe_3_O_4_+*n*C_2_H_4__ is the total energy of the Rh-decorated Fe_3_O_4_(001) surface with adsorbed C_2_H_4_, *E*_Rh/Fe_3_O_4__ is the total energy of the Rh-decorated Fe_3_O_4_(001) surface, *E*_C_2_H_4__ represents the energy of C_2_H_4_ molecule in the gas phase, and *n* is the number of C_2_H_4_ molecules.

## 3 Results

**Figure 1:**
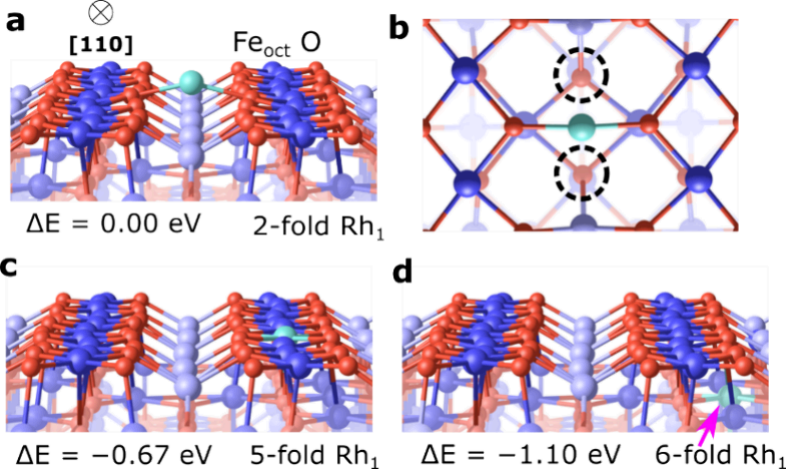
DFT-determined structure models for the C_2_H_4_/Rh_1_/Fe_3_O_4_(001) system. (a) Perspective and (b) top view models of a 2-fold oxygen coordinated Rh_1_ on the Fe_3_O_4_(001) support. The two dashed circles in (b) indicate two equivalent subsurface oxygen atoms in the support, with which the Rh atoms can form a weak bond. (c) A 5-fold coordinated Rh_1_ atom in a substitutional cation site and (d) a subsurface Rh site (pink arrow) with 6-fold coordination to lattice oxygen. Oxygen atoms are red in the models, while surface 5-fold coordinated Fe_oct_ atoms are dark blue. Rh is shown as cyan.

The Rh/Fe_3_O_4_(001) model system has been studied recently by different groups.^15,34−36^ In general, the reconstructed Fe_3_O_4_(001) surface has been shown to provide four possible configurations for Rh atoms: (1) adatom sites with 2-fold coordination to surface oxygen, (2) surface substitutional sites with 5-fold coordination to oxygen, (3) subsurface substitutional sites with 6-fold coordination to oxygen, and (4) Rh clusters, including well-defined dimers.^34^ In Figure 1a–d, we show the DFT-determined structure and energetics for the isolated Rh_1_ geometries. The structure shown in Figure 1a,b is the 2-fold coordinated Rh_1_ adatom. It protrudes 0.7 Å above the O atoms in the surface plane and has a Bader charge of 0.7*e*, and the adsorption energy is calculated to be −4.42 eV, referenced to a gas phase Rh atom. The calculated magnetic moment of 1.91μ_B_ is consistent with a +1 oxidation state for a 2-fold coordinated Rh. This initial geometry is consistent with all other Fe_3_O_4_(001)-based model single-atom catalysts studied to date after deposition at room temperature.^22,23,37^ The 5-fold Rh_1_ shown in Figure 1c can be considered as the substitution of a surface iron cation by Rh. This site is 0.67 eV more stable than the 2-fold Rh_1_, and it exhibits a Bader charge of 1.27*e*. The 6-fold coordinated, subsurface Rh_1_ in a substitutional site, shown in Figure 1d, has an energy 1.10 eV more favorable than the 2-fold Rh_1_ (−5.52 eV with respect to a single gas-phase Rh) and exhibits a Bader charge of 1.24*e*. The magnetic moments are 0.06 (5-fold Rh) and 0.04μ_B_ (6-fold Rh) taking the spill-over of the magnetic moments from the substrate atoms into account, this can be considered a zero magnetic moment as expected for Rh^3+^ with a d^6^ occupation (t_2g_ orbitals occupied, e_g_ empty; the preferred configuration in an octahedral environment). Since the 6-fold Rh is subsurface and fully coordinated to O, it cannot bind to a C_2_H_4_ molecule. In what follows, we primarily consider adsorption at the 2- and 5-fold Rh sites.

**Figure 2:**
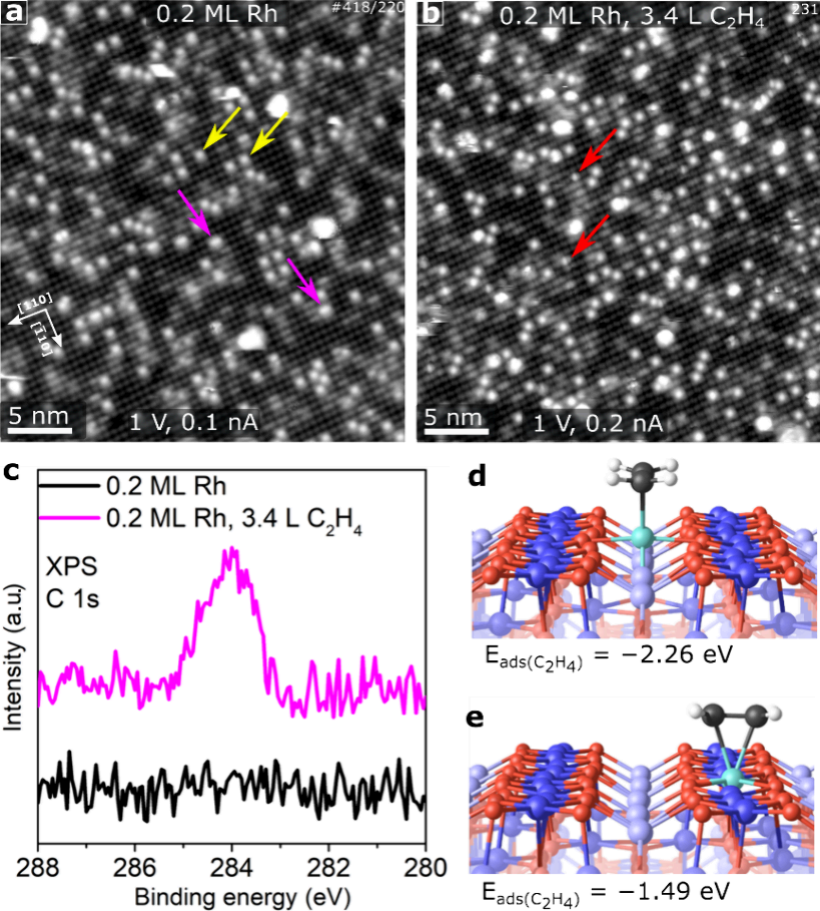
C_2_H_4_adsorption on the Rh_1_/Fe_3_O_4_(001) surface. STM images of the as-prepared 0.2 ML Rh_1_/Fe_3_O_4_(001) surface (a) before and (b) after 3.4 L of C_2_H_4_ adsorption. The yellow arrows indicate 2-fold coordinated Rh_1_ atoms, which are located between the iron rows. The pink arrows indicate Rh dimer species, identified in previous work.^34^ In image (b) acquired at a different position after adsorption of C_2_H_4_, the red arrows indicate protrusions within the surface iron rows, which are due to C_2_H_4_ adsorbed on 5-fold coordinated Rh_1_ atoms. (c) C1s XPS acquired from the 0.2 ML Rh_1_/Fe_3_O_4_(001) sample before (black curve) and after exposure to 3.4 L C_2_H_4_ (pink curve), the curves have been shifted vertically for clarity. (d,e) DFT-derived minimum energy structures for C_2_H_4_ on 2-fold Rh_1_ and 5-fold Rh_1_, respectively. The oxygen atoms are red in the models, while surface 5-fold coordinated Fe_oct_ atoms are dark blue. Rh is shown as cyan. The carbon and hydrogen atoms of the ethylene molecule are colored black and white, respectively.

Figure 2a shows an STM image acquired after Rh was deposited directly on the as-prepared Fe_3_O_4_(001) surface at room temperature via physical vapor deposition (PVD) (see Figure S1 for a corresponding image of the as-prepared surface prior to deposition and details of the typical surface defects observed). The bright rows of protrusions running in the ⟨110⟩-type directions are due to the surface Fe_oct_ atoms, and the bright protrusions (indicated by yellow arrows in Figure 2a) located in-between these rows are assigned to 2-fold coordinated Rh_1_ adatoms. Note that the surface oxygen atoms are not resolved in the images, as they have no density of states in the vicinity of the Fermi level. Nevertheless, their positions are well-known from diffractions-based experiments.^38^ A few small clusters are also observed even at this low coverage, which may be linked to sintering induced by residual O_2_ following sample preparation.^35^ The pink arrows in Figure 2a indicate Rh_2_ dimers, which are slightly extended along the direction of the surface iron rows, different from single atoms (yellow arrows).^34^

In Figure 2b, we show the 0.2 ML Rh_1_/Fe_3_O_4_(001) surface after exposure to 3.4 L of C_2_H_4_ at room temperature. The appearance and apparent height of the protrusions related to the 2-fold Rh are identical to before the exposure within experimental error. Nevertheless, we are confident that these atoms have adsorbed C_2_H_4_ because C 1s XPS data (Figure 2c) obtained from these samples exhibit a peak at ≈ 284 eV consistent with C_2_H_4_ adsorption. Moreover, the area of this peak is approximately double that obtained for a similar coverage of Rh monocarbonyls studied previously.^22,34,35^ This suggests that each Rh_1_ molecule adsorbed a single C_2_H_4_ molecule, which is not visible as a change in the STM contrast. In the prior CO experiment, a weak reduction in the apparent height was observed.^22,34,35^ Interestingly, the density of adatoms located between the surface Fe rows is approximately 23% higher than before the C_2_H_4_ exposure, suggesting that some redispersion of clusters has occurred. We do not observe any species immediately identifiable as the Rh dimer species, so it seems likely that C_2_H_4_ adsorption can break these species apart into 2 adatoms, as was observed previously after CO exposure.^34^ Finally, small bright protrusions directly over the Fe rows (red arrows) are due to adsorption at 5-fold coordinated Rh sites. The coverage of these species is ≈ 12% of the 2-fold Rh coverage.

The minimum-energy configuration obtained computationally for a C_2_H_4_ molecule adsorbed on a 2-fold Rh_1_ species on Fe_3_O_4_(001) is shown in Figure 2d. The carbon–carbon double bond lies parallel to the iron rows, and the adsorption energy is –2.26 eV. An alternative configuration in which the carbon-carbon double bond of C_2_H_4_ lies perpendicular to the iron row (shown in Figure S2) has a weaker adsorption energy of –1.86 eV. The C_2_H_4_ adsorption causes the Rh atom to sink down toward the surface by 0.4 Å, facilitating the formation of a weak bond (≈2.36 Å) between the Rh and a subsurface oxygen atom (the relevant O atoms are highlighted by the dashed black circles in Figure 1b). If one considers the Rh−π interaction as a single ligand, the resulting structure creates a pseudosquare planar environment for the Rh atom. This behavior is identical to that observed recently following CO adsorption, so the analogy appears sound.^34,39^ As in the CO case, the presence of 2 symmetrically equivalent configurations will allow the system to flip rapidly at room temperature as illustrated in Movie S1 (the DFT-determined flipping barrier is ≈0.1 eV). The minimum energy configuration of C_2_H_4_ on a 5-fold Rh_1_ site has the C–C bond perpendicular to the Fe row directions, with an adsorption energy of −1.49 eV (see Figure 2e). This is significantly stronger than for C_2_H_4_ on Fe sites on the pristine surface (DFT result with the same functional: −0.54 eV).^40^

**Figure 3:**
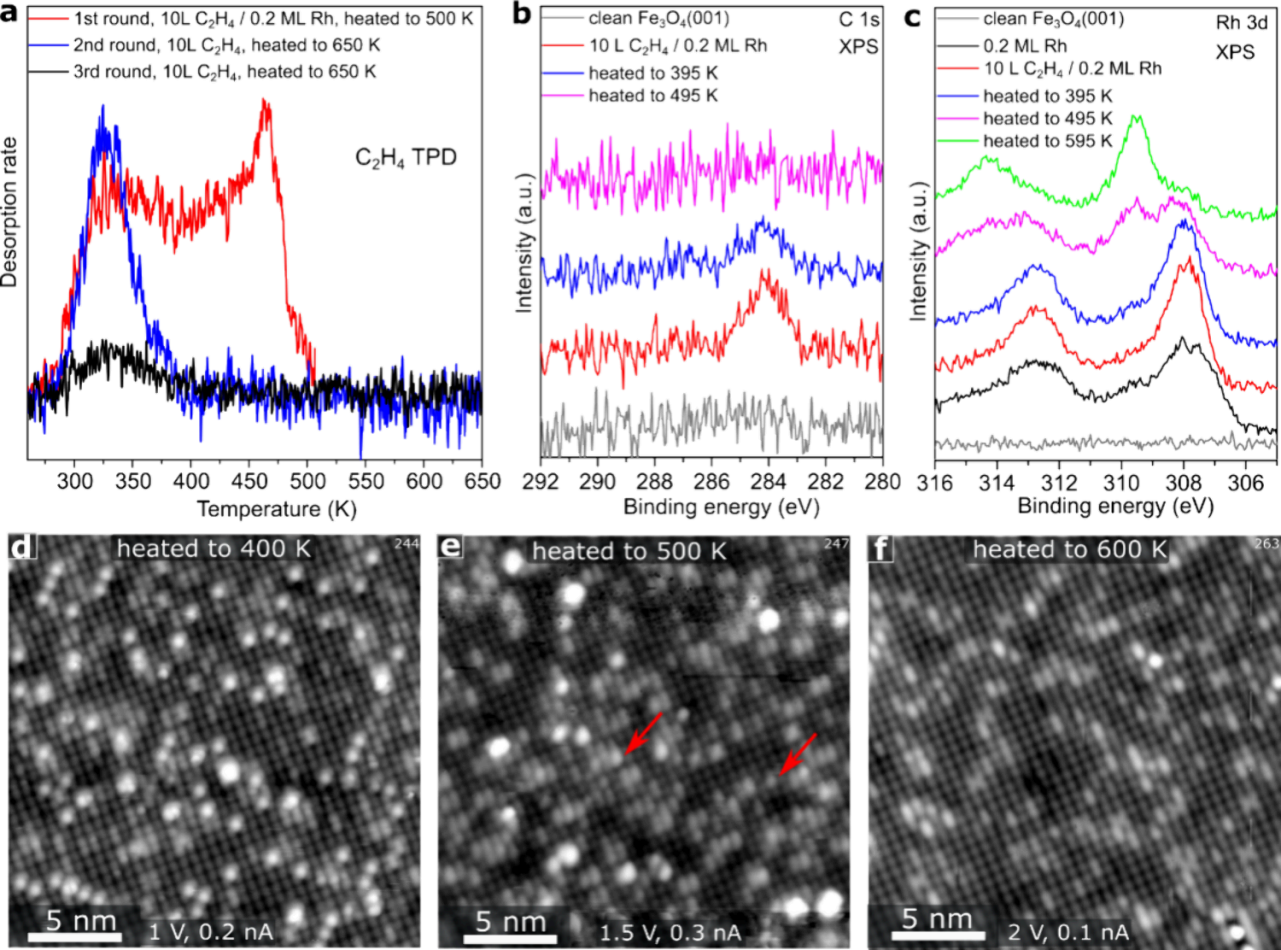
C_2_H_4_ desorption and Rh evolution. (a) A series of C_2_H_4_ TPD spectra obtained from 0.2 ML Rh/Fe_3_O_4_(001) sample following exposure to 10 L C_2_H_4_ at 293 K. The TPD was run from 250 to 500 K in the first round. The sample was then cooled to 293 K, and a further 10 L of C_2_H_4_ adsorbed. In the second and third TPD rounds, the temperature was ramped from 250 to 650 K. (b,c) XPS of C 1s and Rh 3d collected after different flashing temperatures. The spectra are collected after sample cooling down to room temperature. (d–f) STM images of the sample in Figure 2b, followed by annealing at 400, 500, and 600 K. The red arrows in panel (e) indicate 5-fold coordinated Rh_1_, which may have either formed from 2-fold coordinated Rh_1_ during annealing or was present already previously as 5-fold Rh. In any case, it has now lost adsorbed C_2_H_4_. After annealing at 600 K, panel (f) shows that the sample is similar to a clean Fe_3_O_4_(001) surface as shown in Figure S1.

Further evidence of C_2_H_4_ adsorption at the Rh sites comes from TPD and XPS experiments. The red TPD curve in Figure 3 was acquired from a 0.2 ML Rh/Fe_3_O_4_(001) sample after saturation exposure (see Figure S3) at room temperature. The sample was cooled to 250 K prior to starting the TPD ramp. A continuous C_2_H_4_ desorption spectrum is obtained with a clear peak at 462 K. This suggests that desorption occurs from a variety of different sites with different adsorption energies. To gain a comprehensive understanding of the desorption process, we analyzed ethylene and Rh using XPS at specific temperatures within the TPD peak. In Figure 3b, the C 1s XPS data reveal that the area of the C_2_H_4_ peak diminishes by ≈ 1/3 when the sample is heated to approximately 395 K. This aligns well with the desorption curve observed in TPD, as less than half of the ethylene is desorbed at that temperature. After annealing to 495 and 595 K, no carbon species can be detected by C 1s of XPS. We thus conclude that ethylene desorption does not lead to substantial ethylene decomposition and coking of the Rh, and that most, if not all, ethylene desorbed molecularly. This differs from the experience for C_2_H_4_ adsorption and desorption on alumina-supported Pt nanoclusters and a Rh(111) single crystal, where dissociation and coking are observed.^41,42^

Turning now to the Rh 3d region (Figure 3c), the peak is relatively broad for the as-deposited surface as it contains contributions from small clusters (307 eV), 2-fold Rh adatoms (307.7 eV), 5-fold Rh atoms (308.0 eV), and 6-fold atoms (309.5 eV).^35^ The peak becomes sharper after C_2_H_4_ adsorption due to the loss of the low binding energy component. This most likely originates from the redispersion of Rh dimers and small clusters during C_2_H_4_ adsorption, which was hypothesized on the basis of the STM images above. The binding energy shift of Rh 3d caused by C_2_H_4_ adsorption, as determined by DFT calculations for 2-fold Rh, is about 0.2 eV, which is considered negligible. This is close to the experimental error. Such a shift would be hardly noticeable on the scale of Figure 3c. No major change in the chemical state and intensity of the Rh 3d spectrum is apparent upon heating to 395 K (blue curve), despite desorption of a substantial amount of the C_2_H_4_. The main change observable in STM (Figure 3d) is the disappearance of the molecules that were adsorbed at the 5-fold Rh sites. We will show later that it is likely that C_2_H_4_ also desorbed from Rh clusters in this temperature range. Between 395 and 495 K, there is an evolution of the Rh spectrum as the remainder of the ethylene desorbs (Figure 3c, pink curve). The peak splits into two distinct components. The peak at 309.5 eV is attributed to the 6-fold coordinated Rh species, while the peak at 308 eV is due to 5-fold Rh.^35^ This assignment is confirmed by the STM image obtained after annealing at 500 K, in which there are essentially no 2-fold Rh adatoms remaining (Figure 3e). Instead, many new protrusions are detected (red arrows), and these reside within the surface Fe rows and have a similar appearance to the 5-fold coordinated Rh_1_ shown in Figure S4. Some Rh clusters also remain after this annealing step. When the sample is heated further to 600 K, neither 2-fold nor 5-fold Rh remains visible in STM (figure 3f), and the Rh 3d region is dominated by the peak attributed to subsurface 6-fold coordinated Rh atoms (green curve).

In order to further probe the evolution of rhodium species after ethylene desorption, we conducted multiple rounds of C_2_H_4_ TPD experiments as shown in Figure 3a. After the first-round TPD ramp was terminated at 500 K, the sample was cooled to room temperature and re-exposed to C_2_H_4_. Thus, the second TPD curve (shown in blue) is performed on a sample resembling that shown in Figure 3e. In this case, the high-temperature peak is missing, and the only C_2_H_4_ desorption is observed to peak at around 330 K. This is further evidence that the most weakly bound C_2_H_4_ in our experiments is located at the 5-fold Rh_1_ sites and small clusters. A third repeat of the experiment conducted after terminating the previous ramp at 650 K, exhibits a small desorption peak at 330 K, most likely from a few remaining clusters or 5-fold Rh that has not diffused to deeper layers yet. The majority of the Rh atoms have been incorporated into the subsurface layers.

Although the desorption profile is rather broad, the peak at 462 K is sufficiently sharp that it is possible to perform an analysis of the data to extract the adsorption energy of C_2_H_4_ at the 2-fold adatom sites. For this, we utilized a recently developed TPD analysis program described in detail in ref (43) that we had previously used for C_2_H_4_ on the clean Fe_3_O_4_(001) surface.^40^ The key assumption in this work is that the system can be treated as a lattice gas, which essentially means that the molecules do not have any translational degrees of freedom at the desorption temperature. This seems reasonable because the molecules are much more strongly bound at the Rh atom than the surrounding surface, and these sites are clearly still resolved in STM (Figure 3d) after heating to 400 K, which is close to the onset of the desorption peak). With this assumption, we compute an experimental adsorption energy of −1.72 eV. The details of the TPD data analysis are shown in Figure S5 and in Table S2.

## 4 Discussion

The interaction of C_2_H_4_ with a Rh/Fe_3_O_4_(001) model catalyst was studied by using a combination of surface-sensitive techniques and DFT-based calculations. A surprising amount of heterogeneity exists in the initial state of the system considering the apparent homogeneity of the support and the significant energetic differences calculated for the different Rh configurations. The majority of the Rh is initially accommodated in a metastable configuration as 2-fold coordinated Rh adatoms. This suggests kinetic stabilization and thus a barrier to incorporate the Rh into the surface and finally subsurface, where it achieves a higher coordination to oxygen. Nevertheless, some Rh gets incorporated already at room temperature,^35^ which suggests that there are locations on the surface that facilitate easier incorporation of the Rh. It could be that an incoming Rh atom incident at a particular site within the unit cell encounters a lower barrier for incorporation or that the proximity to (sub)surface defects eases the process. Unfortunately, it is not possible to tell from the STM images because Rh incorporation will displace Fe atoms from the subsurface layers where the effect is not visible. However, some insights can be gleaned from a consideration of the thermal evolution of the system. In the absence of C_2_H_4_, annealing the sample to 420 K is sufficient to convert all the 2-fold Rh into more stable configurations, although a mixture of 5- and 6-fold atoms is obtained (Figure S4). C_2_H_4_ adsorption delays the incorporation of 2-fold Rh into the surface. Together with the fact that a significant proportion of the 5-fold signal clearly remains in XPS after heating to 595 K, we conclude that some sites on the surface stabilize the 5-fold configuration more than others. In addition, one may consider a model where sparse defects such as subsurface Fe vacancies may facilitate the incorporation of Rh as soon as the vacancies get mobile.^44^ In such a model, Rh incorporation would depend on whether an Fe vacancy happens to pass under the adsorption site. Thus, the heterogeneity could be partly due to a low concentration of Fe vacancies. Apart from Rh_1_ species with different coordination, we also observe clusters in the initial state, even at low Rh coverage. Their formation could be the result of random chance, i.e., deposited atoms land in the same place during PVD, but it may also be the result of sintering by residual O_2_ that remains in the preparation chamber after sample preparation.^35^

The heterogeneity of the model system is clearly seen in the broad TPD data obtained from the as-prepared surface. Nevertheless, we are able to ascertain that the most strongly bound C_2_H_4_ resides at the 2-fold Rh atoms. The adsorption energy obtained from our TPD analysis (−1.72 eV, see Figure S5) is much lower than the DFT-computed value of −2.26 eV, even when taking into account that the inclusion of dispersion (D3) over binds small molecules by 0.2–0.3 eV.^22,40^ This indicates that the apparent adsorption energy determined from TPD includes the tendency of Rh to assume a higher coordination to oxygen. This suggests that the Rh sheds the C_2_H_4_ in the transition to the 5-fold or 6-fold position. Actually, we saw previously that CO-Ir_1_ could incorporate as a single entity.^39^ Therefore, the desorption energy obtained from TPD analysis is not simply the energy difference between 2-fold coordinated Rh with and without adsorbed C_2_H_4_, but it also includes a contribution from the energy gained upon incorporation of Rh during annealing. The link between adsorption and the Rh site is also evidenced by the fact that the C_2_H_4_ keeps the Rh in 2-fold sites until the 462 K desorption peak is reached, while Rh would otherwise get incorporated into the surface already at 420 K (Figure S4). The adsorption geometry of C_2_H_4_ on 2-fold Rh is very similar to that obtained previously for CO, where the Rh forms a weak bond to a subsurface oxygen resulting in a pseudosquare planar environment for the Rh.^34^ The stability of this environment is in line with the experience of coordination chemistry for Rh(I) systems.

Based on the STM images obtained after heating to 500 K and the appearance of the second TPD round, we conclude that the TPD signal occurring in the 300–400 K temperature regime is due to C_2_H_4_ desorbing from 5-fold Rh sites. This fits with the much lower adsorption energy calculated by DFT (−1.49 eV). Note that we did not specifically analyze the low temperature region because desorption begins immediately at 300 K during the TPD ramp (i.e., the adsorption temperature), which suggests the appearance of the peak in the second TPD round is partly a consequence of the way the experiment was performed. Nevertheless, we conclude that the weakly bound C_2_H_4_ is primarily at the 5-fold Rh sites, and the broadness of the TPD is linked to variations in the environment of the 5-fold Rh (e.g., adjacent defects) as well as the presence of Rh clusters with various sizes. No coking of the system was observed, which could be linked to C_2_H_4_ decomposition at the Rh clusters.

The similarity of the adsorption behavior for C_2_H_4_ and CO extends beyond the pseudosquare-planar structure formed at the 2-fold Rh site. In the CO study, we observed that gem dicarbonyl species could not form directly at a 2-fold Rh site, despite the fact that such a configuration should be thermodynamically stable.^34^ In the present case, a Rh_1_ diethylene structure is also computed to be possible as shown in Figure S6, and would have a similar structure to the gem dicarbonyl. The differential adsorption energy calculated for the second C_2_H_4_ is only −1.02 eV. Considering the overbinding of the DFT calculations, this might be not enough for stable adsorption of the second C_2_H_4_ at room temperature. Nevertheless, we also consider it likely that diethylene formation is prevented by the steric hindrance of the mono C_2_H_4_ species. Interestingly, while Rh gem dicarbonyls could be formed through the decomposition of Rh dimers via a metastable Rh_2_(CO)_3_ configuration,^34^ we do not observe any species attributable to Rh diethylene in the present study. One possible difference between the CO and the C_2_H_4_ case could be that adsorption of 2 ethylene molecules might be sufficient to split the Rh dimers, and a Rh_2_(C_2_H_4_)_3_ intermediate (leading to a gem-diethylene) will never be formed. An alternative explanation is that adsorption of three ethylene molecules on a dimer leads to its dissociation, in analogy to the CO case,^34^ but at room temperature, the resulting Rh(C_2_H_4_)_2_ loses its second ethylene soon, due to the insufficient differential adsorption energy. As found for CO, formation of a diethylene at the 5-fold Rh site is impossible.

The difference in the adsorption energies of CO and C_2_H_4_ has important consequences for performing hydroformylation of alkenes using SACs, as both reactants are supposed to adsorb at the same Rh site. Clearly, CO will poison the catalyst unless the reaction is performed at a temperature where CO will desorb. The formation of gem-dicarbonyls has been observed by diffuse reflectance infrared Fourier transform spectroscopy in most studies,^21,45^ and the reaction is generally performed with a significant excess of C_2_H_4_. The lack of dicarbonyls and diethylene forming in our studies likely means that coadsorption of CO and C_2_H_4_ will also not occur in UHV experiments, which suggests that ambient-pressure studies will be required to shed more light on this important reaction.

## 5 Conclusions

The adsorption of ethylene on a Rh/Fe_3_O_4_(001) model catalyst was investigated by using surface-sensitive techniques and DFT calculations. The adsorption of ethylene induces a downward relaxation of the 2-fold coordinated Rh_1_ and leads to a weak coordination with the subsurface oxygen atoms of Fe_3_O_4_(001). This C_2_H_4_ adsorption (DFT-determined adsorption energy of −2.26 eV) results in the formation of a pseudosquare planar configuration for the Rh atom, as found previously for CO. Adsorption at 5-fold coordinated sites is significantly weaker (−1.49 eV). The TPD spectrum of C_2_H_4_ is broad due to the existence of different Rh species and because C_2_H_4_ desorption from 2-fold Rh_1_ sites occurs in conjunction with the incorporation of the Rh atom into the surface. All ethylene desorption occurs by 500 K, and Rh tends to incorporate in the subsurface layers of the support, where it becomes unavailable for further adsorption.

## Supporting Information Available

STM image of the clean Fe_3_O_4_(001) surface; STM images and XPS data of Rh/Fe_3_O_4_(001) before and after annealing at 420 K; model structures of one and two C_2_H_4_ adsorbed on Rh_1_/Fe_3_O_4_(001); series of C_2_H_4_ TPD results obtained after various C_2_H_4_ exposures at room temperature on a 0.2 ML Rh/Fe_3_O_4_(001); details of analysis of the TPD spectra using the TPD program (PDF)
C_2_H_4_ flipping (AVI)

## Supplementary Material

jp4c03588_si_001.pdf

jp4c03588_si_002.avi
