# Supplementary material for: A Multitechnique Study of C2H4 Adsorption on a Model Single-Atom Rh1 Catalyst
Source: J Phys Chem C Nanomater Interfaces. 2024 Sep 5;128(37):15404–11. doi: 10.1021/acs.jpcc.4c03588 (PMC11421075; doi:10.1021/acs.jpcc.4c03588)
Supplement: Supplementary file 1 — jp4c03588_si_001.pdf [file jp4c03588_si_001.pdf]

Supplementary Information:

A Multitechnique Study of C_2H_4 Adsorption on a Model Single-Atom Rh_1 Catalyst

Chunlei Wang [1]*, Panukorn Sombut[1], Lena Puntscher[1], Manuel Ulreich[1], Jiri Pavelec[1], David Rath[1], Jan Balajka[1], Matthias Meier[1,2], Michael Schmid[1], Ulrike Diebold[1], Cesare Franchini[2,3], and Gareth S. Parkinson[1]

[1]Institute of Applied Physics, TU Wien, Vienna, Austria

[2]Faculty of Physics, Center for Computational Materials Science, University of Vienna, Vienna, Austria

[3]Dipartimento di Fisica e Astronomia, Università di Bologna, Bologna, Italy

The PDF file includes:

Table S1

Figures S1 to S5

Table S2

Figure S6

Other Supplementary Material for this manuscript includes the following:

Movie S1: C_2H_4 flipping

Tables S1. Comparison of the adsorption energies using the PBE+U+D3(BJ) and PBE0+D3(BJ) functionals for C_2H_4 adsorption on 2-fold and 5-fold oxygen coordinated Rh adatoms supported by Fe_3O_4(001). Note that the unit cell is smaller than that used for the other calculations, leading to slightly different values. The difference of the adsorption energy between the two different configurations is comparable for both functionals (0.58 vs. 0.65 eV).

	PBE+U+D3(BJ)	PBE0+D3(BJ)
C_2H_4/2-fold Rh	−2.24 eV	−2.08 eV
C_2H_4/5-fold Rh	−1.66 eV	−1.43 eV

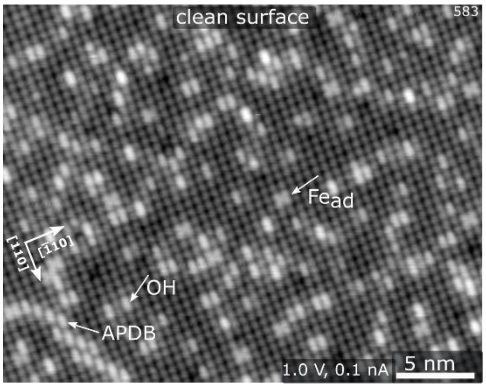

Figure S1. The as-prepared Fe_3O_4(001) clean surface. Scanning tunneling microscopy (STM) image of the reconstructed Fe_3O_4(001) surface prepared by cycles of sputtering and annealing in a partial pressure of 2×10^{-6} mbar O_2. The protrusions forming rows in [110] direction are due to pairs of surface Fe atoms. Bright protrusions are related to various surface defects including a surface hydroxyl group (OH), and anti-phase domain boundary in the surface reconstruction (APDB) and an Fe adatom Fe_{ad}.[1] Each is labelled within the image.

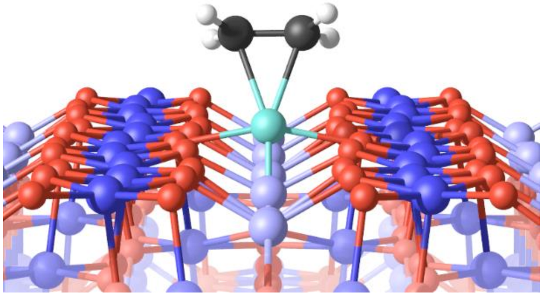

Figure S2. An alternative variant of C_2H_4 adsorption on the 2-fold coordinated Rh_1 determined by DFT calculation. In this variant, the C=C double bond of C_2H_4 molecule is perpendicular to the iron rows of $Fe_3O_4(001)$ support. The adsorption energy of C_2H_4 towards this structure is –1.86 eV, which is less favorable compared to the C_2H_4 adsorption structure in Figure 2d, which features a C=C bond parallel to the surface Fe rows. The oxygen atoms are red in the models, while surface 5-fold coordinated Fe_{oct} atoms are dark blue. Rh is shown as cyan. The carbon and hydrogen atoms of the ethylene molecule are shown black and white, respectively.

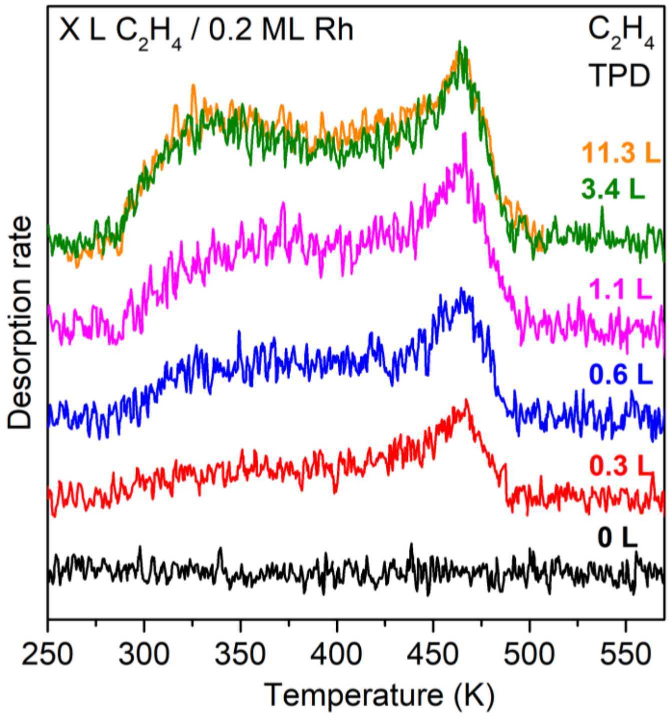

Figure S3. A series of C_2H_4 TPD results obtained after various C_2H_4 exposures at room temperature on a 0.2 ML $Rh/Fe_3O_4(001)$ sample (gas doses, X = 0, 0.3, 0.6, 1.1, 3.4, 11.3 Langmuir). As the dosage of C_2H_4 increases, the C_2H_4 desorption peaks saturate at 3.4 L C_2H_4. This is demonstrated by overlapping the spectra for 3.4 L and a larger dose of 11.3 L C_2H_4 at the top of the figure.

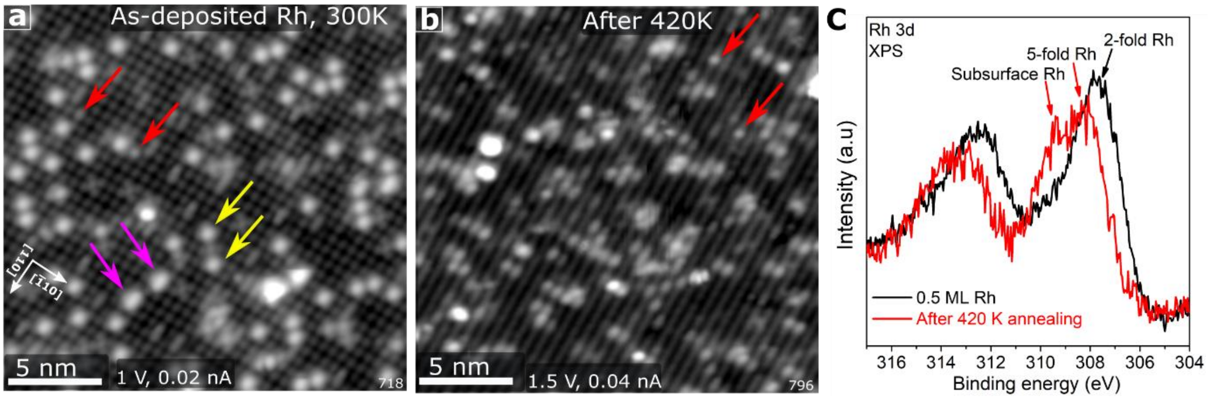

Figure S4. Thermal stability of the as-prepared model single-atom $Rh_1/Fe_3O_4(001)$ catalyst. STM images of (a) 0.2 ML Rh on $Fe_3O_4(001)$ after Rh deposition at 300 K and (b) after annealing at 420 K. The yellow arrows indicate 2-fold coordinated Rh_1 atoms, which are located in-between the surface Fe rows. The red arrows indicate 5-fold coordinated Rh_1 atoms located in the Fe rows along [110] direction. The pink arrows indicate Rh dimer species. (c) XPS Rh 3d collected on the as-prepared Rh sample (black curve) and after annealing at 420 K (red curve). The XPS binding positions of 2-fold Rh, 5-fold Rh, and 6-fold Rh (Rh in subsurface) are pointed out by the black and red arrows.[2] At first glance, it may seem surprising that the change of the core level energies between the 2-fold and 5-fold coordination is less than that between 5-fold and 6-fold coordination. It has to be noted, however, that Rh core level energies do not always follow simple rules, oxygen neighbors can even cause a shift to lower binding energies than in metallic bulk Rh.[3,4]

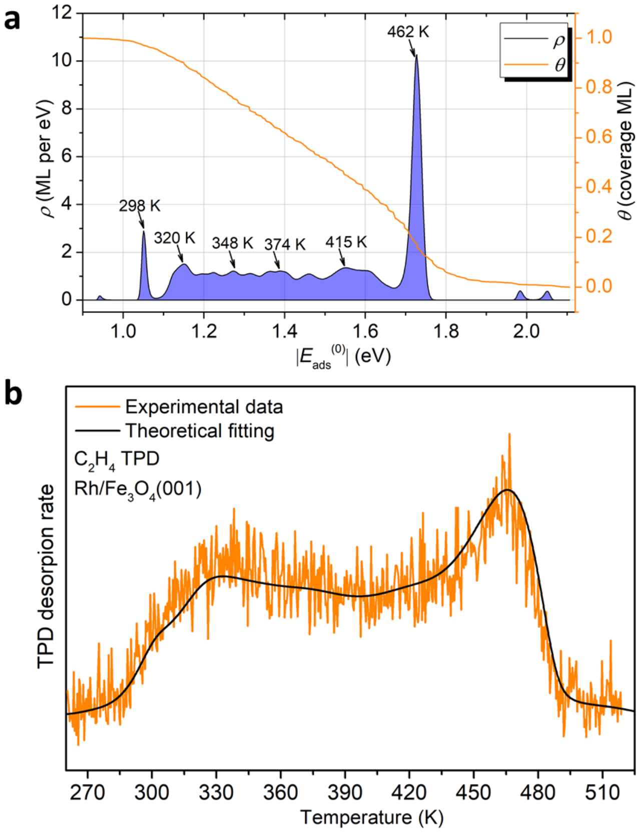

Figure S5. Analysis of the TPD spectra using the TPD program introduced recently.[5] (a) Distribution of the adsorption energies derived from the experimental C_2H_4 TPD spectra (orange curve in b). The orange curve in (a) shows the fraction of molecules with stronger adsorption than the given value on the x-axis, e.g., the onset of the peak at 462 K is at a coverage between 0.25 and 0.3 of the initial (saturation) coverage. The temperatures in (a) provide a rough indication of the correspondence of the peaks and wiggles in the adsorption energy distribution with features in the spectrum. (b) TPD spectra calculated from the adsorption energy distribution (black curve) in panel (a) plotted on top of the experimental data (orange). The analysis assumes is based on the parameters in Table S1 and results in an adsorption energy of −1.72 eV for the peak at 462 K. A conventional Polanyi–Wigner TPD analysis would yield this result when assuming a pre-exponential factor of 2×10^{18} s^{-1}.

Table S2: Input parameters for the TPD analysis

C_2H_4 gas, atomic coordinates (Å)	C	0.6579	−0.0045	0.0639
	H	1.1610	0.0661	1.0238
	H	1.3352	−0.0830	−0.7815
	C	−0.6579	0.0045	−0.0639
	H	−1.3355	0.0830	0.7812
	H	−1.1608	−0.0661	−1.0239

Heating ramp β	1 K/s	
Areal density of adsorption sites n_a	2.81×10^{17} per m^2 (0.2 ML Rh)	
Initial coverage θ_0	1	
Vibration frequencies [a]	C_2H_4	9.57, 17.72, 20.37, 49.50, 60.88, 85.09 meV
	Rh	18.78, 30.30, 38.47, −10.04, −12.85, −37.71 meV
Extra entropy [b]	2 k_B	
Langmuirian initial sticking s_0	0.06 [c]	

[a]Frequencies from DFT. Vibrations only present on the bare surface (without the adsorbate) must be entered as negative numbers in the program. The vibrations influence the calculated adsorption energy through the vibrational entropy; omitting the vibrations would lead to an overestimation of the magnitude of the adsorption energy by 0.12 eV.

[b] Two equivalent configurations due to C_2H_4 flipping on $Rh_1/Fe_3O_4(001)$.

[c] This value corresponds to a sticking cross section of 20 Å² per unoccupied Rh atom. This parameter, which describes the sticking at the desorption temperature, leads to the largest uncertainty of the TPD analysis of ±0.1 eV (assuming an uncertainty by ± one order of magnitude). The appearance of the coverage-dependent TPD curve in Fig. S3 indicates that long-distance adsorbate diffusion on the bare Fe_3O_4 surface cannot occur; otherwise, the low-coverage spectra should show desorption only at the high-temperature peak. Long-distance diffusion over the Fe_3O_4 surface would lead to a high sticking coefficient, because the molecules could arrive anywhere on the surface, diffuse and spill over onto the Rh_1 adatoms.

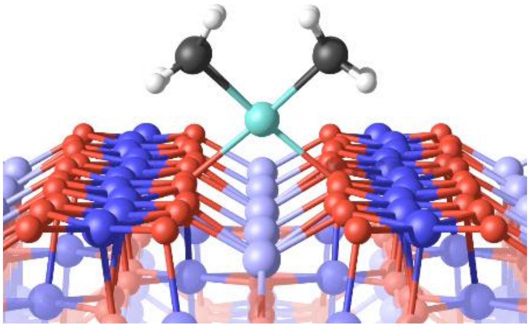

Figure S6. $2C_2H_4$ adsorption on a 2-fold oxygen coordinated Rh_1 calculated by DFT. The average adsorption energy per C_2H_4 molecule is −1.64 eV.

References

(1) R Bliem; E McDermott; P Ferstl; M Setvin; O Gamba; J Pavelec; MA Schneider; M Schmid; U Diebold; P Blaha. Subsurface cation vacancy stabilization of the magnetite (001) surface. *Science* **2014**, *346*, 1215-1218.

(2) Zdenek Jakub; Jan Hulva; Paul TP Ryan; David A Duncan; David J Payne; Roland Bliem; Manuel Ulreich; Patrick Hofegger; Florian Kraushofer; Matthias Meier. Adsorbate-induced structural evolution changes the mechanism of CO oxidation on a Rh/Fe_3O_4(001) model catalyst. *Nanoscale* **2020**, *12*, 5866-5875.

(3) L Köhler; G Kresse; Michael Schmid; Edvin Lundgren; Johan Gustafson; Anders Mikkelsen; Mikael Borg; J Yuhara; Jesper N Andersen; M Marsman. High-Coverage Oxygen Structures on Rh(111): Adsorbate Repulsion and Site Preference Is Not Enough. *Physical Review Letters* **2004**, *93*, 266103.

(4) Johan Gustafson; Anders Mikkelsen; Mikael Borg; Edvin Lundgren; L Köhler; G Kresse; Michael Schmid; Peter Varga; J Yuhara; X Torrelles. Self-limited growth of a thin oxide layer on Rh (111). *Physical Review Letters* **2004**, *92*, 126102.

(5) Michael Schmid; Gareth S Parkinson; Ulrike Diebold. Analysis of temperature-programmed desorption via equilibrium thermodynamics. *ACS Physical Chemistry Au* **2022**, *3*, 44-62.
